# Cortical laminar resting‐state signal fluctuations scale with the hypercapnic blood oxygenation level‐dependent response

**DOI:** 10.1002/hbm.24926

**Published:** 2020-01-20

**Authors:** Maria Guidi, Laurentius Huber, Leonie Lampe, Alberto Merola, Kristin Ihle, Harald E. Möller

**Affiliations:** ^1^ Max Planck Institute for Human Cognitive and Brain Sciences Leipzig Germany; ^2^ Department of Cognitive Neuroscience, Faculty of Psychology and Neuroscience Maastricht University Maastricht Netherlands

**Keywords:** 7 T‐fMRI, calibrated fMRI, hypercapnia, laminar fMRI, resting‐state fMRI, VASO

## Abstract

Calibrated functional magnetic resonance imaging can remove unwanted sources of signal variability in the blood oxygenation level‐dependent (BOLD) response. This is achieved by scaling, using information from a perfusion‐sensitive scan during a purely vascular challenge, typically induced by a gas manipulation or a breath‐hold task. In this work, we seek for a validation of the use of the resting‐state fluctuation amplitude (RSFA) as a scaling factor to remove vascular contributions from the BOLD response. Given the peculiarity of depth‐dependent vascularization in gray matter, BOLD and vascular space occupancy (VASO) data were acquired at submillimeter resolution and averaged across cortical laminae. RSFA from the primary motor cortex was, thus, compared to the amplitude of hypercapnia‐induced signal changes (tSD_hc_) and with the *M* factor of the Davis model on a laminar level. High linear correlations were observed for RSFA and tSD_hc_ (*R*^2^ = 0.92 ± 0.06) and somewhat reduced for RSFA and *M* (*R*^2^ = 0.62 ± 0.19). Laminar profiles of RSFA‐normalized BOLD signal changes yielded good agreement with corresponding VASO profiles. Overall, this suggests that RSFA contains strong vascular components and is also modulated by baseline quantities contained in the *M* factor. We conclude that RSFA may replace the scaling factor tSD_hc_ for normalizing the laminar BOLD response.

**Abbreviations**
3D: three‐dimensional
ALFF: amplitude of low‐frequency fluctuations
ASL: arterial spin labeling
ATP: adenonsine triphosphate
BOLD: blood oxygenation level‐dependent
CSF: cerebrospinal fluid
dHb: deoxyhemoglobin
EPI: Echo‐Planar Imaging
fALFF: fractional ALFF
fMRI: functional MRI
FOV: field of view
GM: gray matter
GRAPPA: GeneRalized Autocalibrating Partially Parallel Acquisitions
GRE: gradient‐recalled echo
M1: primary motor cortex
MP2RAGE: Magnetization Prepared 2 RApid Gradient Echoes
MRI: magnetic resonance imaging
RF: radiofrequency
ROI: region of interest
RSFA: resting‐state fluctuation amplitude
SS‐SI‐VASO: Slice‐Saturation Slab‐Inversion VASO
V1: primary visual cortex
VASO: VAscular Space Occupancy
WM: white matter
*Mathematical*: *symbols*
CBF: cerebral blood flow
CBV: cerebral blood volume
CMR_O2_: cerebral metabolic rate of oxygen consumption
CVR: cerebrovascular reactivity
*f*: normalized CBF
*M*: calibration constant corresponding to the maximal BOLD signal change
P_ET_CO_2_: end‐tidal partial pressure of carbon dioxide
P_ET_O_2_: end‐tidal partial pressure of oxygen
*R*^2^: coefficient of determination
$R_{2}^{\prime }$: reversible transverse relaxation rate
RSFA: fluctuation amplitude of the resting‐state timeseries
*r*: normalized CMR_O2_
*S*: signal amplitude
Δ*S*: relative BOLD signal change
Δ*S*_hc_: relative BOLD signal change induced by mild hypercapnia
*T*_1_: longitudinal relaxation time
TE: echo time
TI: inversion time
TR: repetition time
tSD_hc_: temporal standard deviation of the hypercapnia timeseries
tSD_rs_: temporal standard deviation of the resting‐state timeseries
tSNR: temporal signal‐to‐noise ratio
*p*: error probability
*v*: normalized CBV
*Z*: *Z*‐score
*α*: Grubb exponent
*β*: constant describing the coupling between $R_{2}^{\prime }$ and [dHb]
*κ*: proportionality constant
_0_: index indicating a baseline (“*resting*”) level
_full_: index indicating the entire frequency band
_high_: index indicating the high‐frequency band
_low_: index indicating the low‐frequency band
_*t*_: index indicating the total blood compartment
_*v*_: index indicating the venous compartment
[X]: concentration of compound X

## 1 INTRODUCTION

Functional magnetic resonance imaging (fMRI) based on echo‐planar imaging (EPI) with gradient‐recalled echoes (GRE) is currently the most widespread technique to look at human brain function. However, its baseline‐dependent nature makes data comparisons across participants, brain areas, and brain states challenging. Apart from true differences in neuronal activity, significant differences in the blood oxygenation level‐dependent (BOLD) responses could be attributable to factors that are difficult to control for, such as caffeine intake, time of the day, age, baseline oxygenation, vascularization density, or baseline cerebral blood flow (CBF) (Krieger et al., 2014; Whittaker, Driver, Bright, & Murphy, 2016). The spatial signature of these effects becomes particularly clear at laminar resolutions (Polimeni, Fischl, Greve, & Wald, 2010; Yen, Zhao, & Kim, 2012), such that the so‐called physiological noise (Krüger & Glover, 2001) is strongly linked to the baseline cerebral blood volume (CBV) (Koopmans, Barth, Orzada, & Norris, 2011).

Calibrated fMRI attempts to solve this issue by scaling the task‐related BOLD response by one obtained with a purely vascular challenge, typically hypercapnia. Combined with a biophysical model, this has been used to extract information on (relative) changes of the cerebral metabolic rate of oxygen consumption (CMR_O2_) (Davis, Kwong, Weisskoff, & Rosen, 1998; Gauthier & Hoge, 2013; Hoge et al., 1999). This normalization is believed to result in a quantity that is more directly coupled to the neuronal response than unscaled BOLD signal changes. The underlying rationale is that the oxygen uptake needed for adenosine triphosphate (ATP) production happens at the closest capillary bed. Consequently, it should be more spatially specific to the site of neural activity than the BOLD signal changes, which are driven by the venous vasculature (Huber, Uludağ, & Möller, 2019). The method has proven to account for a high degree of variability in the BOLD response and, therefore, found some application (Blockley, Griffeth, Simon, & Buxton, 2013; Pike, 2012).

Most calibrated fMRI studies suffer from two main drawbacks: (a) They rely on a gas manipulation scan (apart from the functional paradigm). (b) They require the acquisition of an additional non‐BOLD functional contrast for quantifying the vascular response. The vascular challenge has been traditionally taken to be a gas manipulation inducing hypercapnia, under the assumption that it does not induce changes in oxidative metabolism. This isometabolic assumption has not yet been robustly proven, and previous studies have reported a decrease (Xu et al., 2011; Zappe, Uludağ, Oeltermann, Uğurbil, & Logothetis, 2008), an increase (Jones, Berwick, Hewson‐Stoate, Gias, & Mayhew, 2005), or no change (Chen & Pike, 2010; Jain et al., 2011) in CMR_O2_. The metabolic effect of hypercapnia is believed to be dependent on the carbon dioxide (CO_2_) content of the inspired gas, with higher concentration inducing higher oxidative metabolism changes (Jones et al., 2005; Zappe et al., 2008). The setup needed for the gas manipulation usually involves a facial mask or a mouth piece and a nasal clip, which causes discomfort to the participant. Additionally, the inflow of gas may be perceived as unpleasant (e.g., a dry mouth and throat) and may lead to dizziness if the CO_2_ concentration of the gas mixture is high (e.g., above 5%). Such setup‐specific problems can be circumvented by using a breath‐hold task (Kastrup, Li, Glover, & Moseley, 1999). However, this comes at the expense of reduced signal quality (e.g., due to enhanced task‐related head motion) and reproducibility (because of a reduced response amplitude). Moreover, respiratory manipulations might be inapplicable in noncompliant subject populations, such as children, or might be too demanding for patients with pulmonary or cardiac disease (Moreton, Dani, Goutcher, O'Hare, & Muir, 2016) or for elderlies. Finally, the additionally required functional contrast, such as CBF recorded with arterial spin labeling (ASL) (Alsop et al., 2015; Detre, Leigh, Williams, & Koretsky, 1992; Lorenz, Mildner, Schlumm, & Möller, 2018; Mildner et al., 2003) or CBV recorded with vascular space occupancy (VASO) techniques (Hua, Jones, Qin, & van Zijl, 2013; Huber et al., 2014b; Lu, Golay, Pekar, & van Zijl, 2003; Lu, Hua, & van Zijl, 2013), is penalized by a reduced sensitivity compared to the GRE‐BOLD response. Consequently, this has a crucial impact on the quality of the CMR_O2_ estimation (Huber et al., 2019).

Given these obstacles, measures obtained with so‐called resting‐state fMRI have been proposed as alternative normalization approaches. At present, there is, however, no general consensus on how to best extract a scaling factor from the resting‐state time series. Previous attempts include the use of the resting‐state fluctuation amplitude (RSFA) (Kannurpatti & Biswal, 2008), the low‐frequency spectral amplitude (Biswal, Kannurpatti, & Rypma, 2007), the amplitude of low‐frequency fluctuations (ALFF) (Zang et al., 2007), the fractional amplitude of low‐frequency fluctuations (fALFF) (Zou et al., 2008), the power in the low frequencies of the residuals in the task general linear model (Kazan et al., 2017), the temporal correspondence of global low frequency fluctuations with individual voxels (Liu et al., 2017), and others (Golestani, Wie, & Chen, 2016; Jahanian et al., 2017; Liu, 2013; Liu et al., 2013).

Nevertheless, most of the calibrated BOLD studies still rely on gas manipulation or breath‐hold scans rather than on resting state‐based approaches. This might be due to the fact that several different scaling factors have been proposed and most of them are missing a clear validation. Given that the band of interest is the low‐frequency one, which is considered to be more closely coupled to spontaneous neural activity and, thus, used for connectivity studies (Birn, 2012; Murphy, Birn, & Bandettini, 2013), a mixture of neuronal and vascular contributions is expected. This “confounding” neural contribution represents one of the biggest impediments to the use of resting‐state fluctuations as a scaling factor (Lipp, Murphy, Caseras, & Wise, 2015; Liu, 2013). Moreover, the choice of the repetition time, TR, impacts the amount of aliased cardiac and respiration frequencies into the low‐frequency band (Viessmann, Möller, & Jezzard, 2017; Viessmann, Möller, & Jezzard, 2019; Wise, Ide, Poulin, & Tracey, 2004).

So far, these methods have only been applied at relatively low spatial resolution. Their applicability to high‐resolution fMRI, as required for obtaining depth‐dependent information, has not yet been investigated. An increase in resolution is limited, in general, by the achieved temporal signal‐to‐noise ratio (tSNR). A more specific potential problem is related to the interpretation of the BOLD response because traditional assumptions about neurovascular coupling mechanisms may not be valid locally on a submillimeter spatial scale. Voxels of a dimension on the order of the cortical thickness (or above) in a given brain region contain similar mixtures of arterial, capillary, and venous blood. On this spatial scale, it can be assumed that BOLD signal changes are modulated by consistent dynamic changes in CBF, CBV, and CMR_O2_ (Buxton, Uludağ, Dubowitz, & Liu, 2004). On a submillimeter scale, however, different voxels of the same brain region may have different total CBV fractions or different portions of arterial, capillary, and venous blood. For example, voxels located at the pial surface should contain relatively large fractions of arterial and venous blood but should be mostly devoid of capillary blood. In such voxels, significant changes in the deoxyhemoglobin concentration, [dHb], may be observed if they contain veins draining an (upstream) activated area (Markuerkiaga, Barth, & Norris, 2016). However, this local BOLD signal change may not have a colocalized CMR_O2_ change.

One goal of the current work was to investigate the use of RSFA indices for normalizing BOLD signal changes recorded at submillimeter spatial resolution on a laminar basis. This was done by comparing the obtained results to other metrics recorded with gas‐calibration experiments in a separate session. Given that the human neocortex is organized into six layers, each of which can be treated—to some extent—as a functional unit, averaging was performed along laminae rather than across the full patch of cortex. Compared to standard‐resolution scans, laminar fMRI yields additional information about an approximate location within the cortical ribbon where BOLD signal changes occur. This helps to better disentangle contributions from the capillary bed and those from the downstream vasculature (Huber et al., 2015; Kim & Kim, 2010; Koopmans, Barth, & Norris, 2010). For further validation, a second goal was a direct comparison of laminar CBV changes and simultaneously recorded BOLD signal changes with and without RSFA‐based normalization.

## 2 METHODS

### 2.1 Comparison of RSFA‐based normalization and gas calibration

#### 2.1.1 Participants

Ten right‐handed healthy volunteers (6 males, mean age: 256 ± 4 years) with no history of neurological disorders participated in the first part of the study after giving informed written consent. The experimental procedures had been previously approved by the Ethics Committee at the Medical Faculty of the University of Leipzig (Reg.‐No. 273‐14‐25082014). Participants were asked to refrain from coffee and alcohol intake on the day of the experiment. A physician was present during each session to monitor physiological recordings during the breathing manipulation task.

#### 2.1.2 Experimental setup

Magnetic resonance imaging (MRI) was performed on a MAGNETOM 7T scanner (Siemens Healthcare, Erlangen, Germany) using a circularly polarized transmit/32‐channel receive radiofrequency (RF) head coil (Nova Medical, Wilmington, MA).

The gas mixture used to induce hypercapnia contained 5% CO_2_, 21% oxygen (O_2_), and 74% nitrogen (N_2_). It was delivered to the participant through a mouthpiece connected by a tube to a gas wall socket. The mouthpiece was adjusted to fit tightly to the participant's face in order to avoid inflow of room air. A nose clip was used to prevent breathing from the nasal cavity. The inflow of gas was manually adjusted to a rate of approximately 18 L/min (standard temperature and pressure). Normocapnia was restored by disconnecting the tube from the wall socket, thereby letting room air flow into the tube. A second tube connected to the mouth piece was used for directing the exhaled air outside the scanner bore. Inhaled and exhaled gases were prevented from mixing via valves placed in the interior of the mouth piece. Heart beat and respiration timecourses were recorded using the scanner's physiological monitoring unit (pulse sensor and respiratory belt). The end‐tidal partial pressures of CO_2_ (P_ET_CO_2_) and O_2_ (P_ET_O_2_) were recorded using an MP150 system (BIOPAC Systems, Goleta, CA). Written verbal instructions were projected onto a screen, which could be viewed by the participant via a mirror mounted on the RF coil. In case of visual deficiencies, MRI‐compatible glasses were provided to the participant.

#### 2.1.3 Functional paradigm

The paradigm consisted of two sessions, each 15 min long. The first session was a block‐design hypercapnia task, followed by a resting‐state session. The hypercapnia task consisted of an alternation of breathing room air and the CO_2_‐enriched gas mixture. After an initial 2‐min block of room air, the gas mixture was administered for two 3‐min blocks, separated by an equally long block of room air. A final 4‐min block of room air breathing was added for baseline localization. For the resting‐state scan, a fixation image was projected onto the screen, and the participants were asked either to fixate it or to stay with the eyes closed for the whole duration of the experiment and not to fall asleep.

#### 2.1.4 Imaging pulse sequence

Previously acquired MP2RAGE (magnetization prepared two rapid gradient echoes) (Marques et al., 2010) images were inspected in order to localize the primary motor cortex (M1) of each individual participant. Positioning the three‐dimensional (3D) slab for fMRI followed previously established procedures (Guidi, Huber, Lampe, Gauthier, & Möller, 2016; Huber et al., 2015; Huber et al., 2018). In particular, a slice orientation perpendicular to the cortical surface achieves a sufficiently high resolution along the cortical depth while the slice thickness can be increased depending on the individual anatomy to improve the SNR. However, even with a sufficiently wide slab, it is typically not possible to align the slices perpendicularly to M1 on both sides at the same time. Therefore, slice positioning was always optimized for the hand knob area of the left M1, which was exclusively selected for the subsequent data analysis. A slice‐saturation slab‐inversion (SS‐SI) VASO sequence (Huber et al., 2014b) with a hybrid 3D EPI readout (Poser, Koopmans, Witzel, Wald, & Barth, 2010) was used for the acquisition. Previous work has shown that the 3D readout is beneficial for sub‐millimeter applications (Huber et al., 2018). The pulse sequence parameters included an inversion time, TI = 900 ms; an effective repetition time, TR = 1,648 ms; an echo time, TE = 24 ms; a bandwidth of 1,042 Hz/px; a GRAPPA (generalized autocalibrating partially parallel acquisition) (Griswold et al., 2002) acceleration factor of 2; a partial Fourier factor of 6/8; and an asymmetric field of view (FOV) with a matrix size of 132 × 44 × 10 yielding a nominal voxel size of 0.8 × 0.8 × 1.8 mm^3^. Variable flip angles were used to minimize *T*_1_‐related blurring along the slice direction (Huber et al., 2018).

#### 2.1.5 Image preprocessing and layering

All timeseries were corrected for rigid‐body motion using the function “Realign: Estimate and Reslice” in SPM12 (http://www.fil.ion.ucl.ac.uk/spm/). The recorded respiratory and cardiac traces were used for denoising the resting‐state timecourses using the Analysis of Functional NeuroImages (AFNI) (Cox, 1996) implementation of RETROICOR (Glover, Li, & Ress, 2000). All fMRI timeseries were linearly detrended to remove low‐frequency signal drifts.

**Figure 1:**
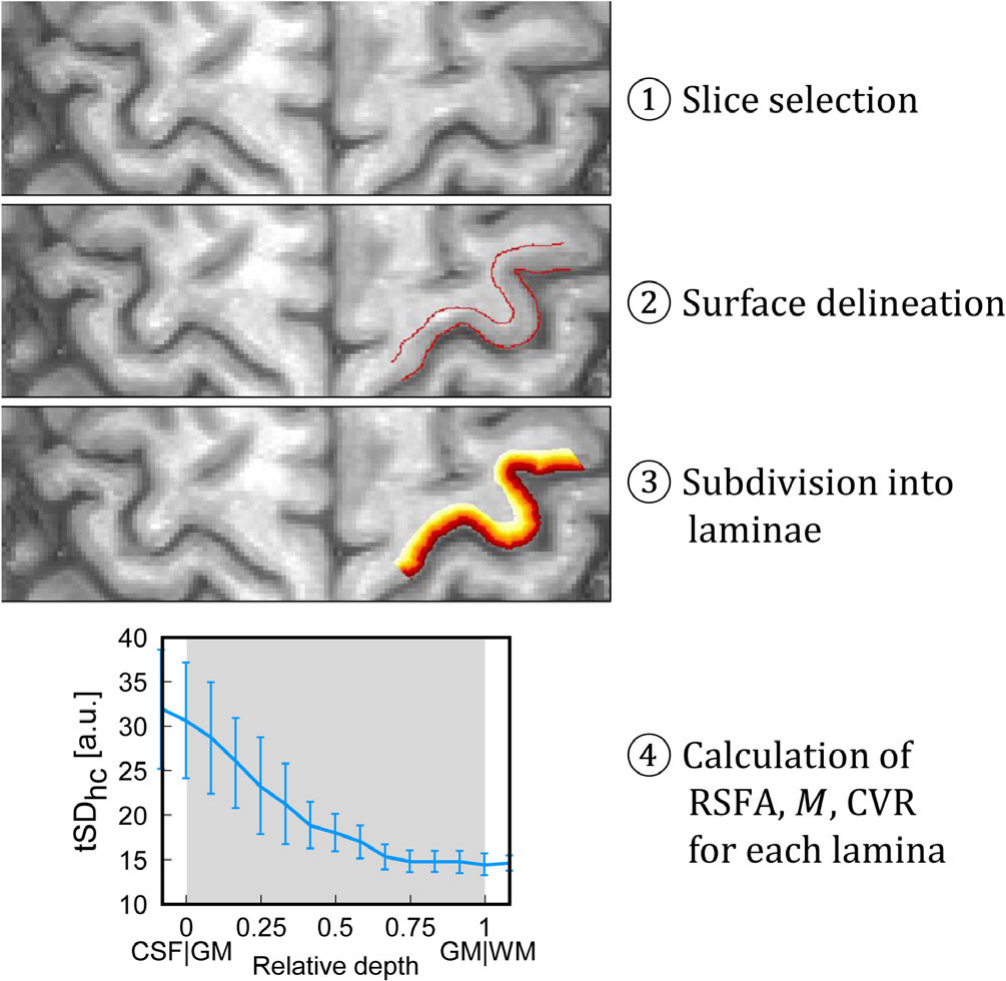
Schematic illustrating the four steps involved in the calculation of depth‐dependent profiles. The slice with the best through‐plane orientation was chosen (1), and the gray matter (GM)/cerebrospinal fluid (CSF) surface and the gray matter (GM)/white matter (WM) surface were manually delineated (2). Equivolume laminae were then generated between these two borders (3). Finally, the quantities of interest were averaged along each individual lamina and plotted as a function of depth (4). Here, the laminar profile of tSD_hc_ measured in a representative participant is shown as an example

Anatomical references for layering were obtained from resized *T*_1_‐weighted EPI maps generated from the functional gas‐manipulation and resting‐state timeseries. The *T*_1_‐weighting was derived from the original SS‐SI‐VASO timeseries consisting of alternating EPI slabs acquired with and without a preceding inversion pulse, that is, prior to the CBV/BOLD contrast splitting (Guidi et al., 2016). Since the signal difference of the interleaved *T*_1_‐weighted VASO and BOLD acquisitions is dominated by longitudinal relaxation, this “anatomical” gray matter (GM)/cerebrospinal fluid (CSF) contrast in EPI space is comparable to the contrast on *T*_1_‐weighted MP2RAGE uniform (“UNI”) images. A representative example is shown in Figure 1.

For layering purposes, the original in‐plane matrix size of 132 × 44 was resized to 528 × 176. Consistent with most segmentation software packages, this upsampling was done to obtain smooth laminae without angularity limitations in voxel space. Gray matter/CSF and GM/white matter (WM) borders were then manually drawn on the slice with the best through‐plane orientation. The region of interest (ROI) was defined as the portion of GM that was (a) well aligned to the slice orientation across the cortical depth and that (b) additionally showed sufficient activation on BOLD and VASO maps. In this ROI, 15 laminae were grown as in previous work (Guidi et al., 2016; Huber et al., 2015) employing C++ and Object‐oriented Development Interface for NMR (ODIN) libraries (Jochimsen & von Mengershausen, 2004). Briefly, the algorithm follows an equivolume approach that preserves the volume fraction of each lamina in cortical segments (Figure 1) and accurately resembles the arrangement of anatomical layers (Waehnert et al., 2014). Throughout this work, we use the term *“laminae”* rather than *“layers”* to stress the fact that they correspond to certain depths from the cortical surface, whereas they do not a priori correspond to single histological layers.

#### 2.1.6 Quantitative analyses

**Figure 2:**
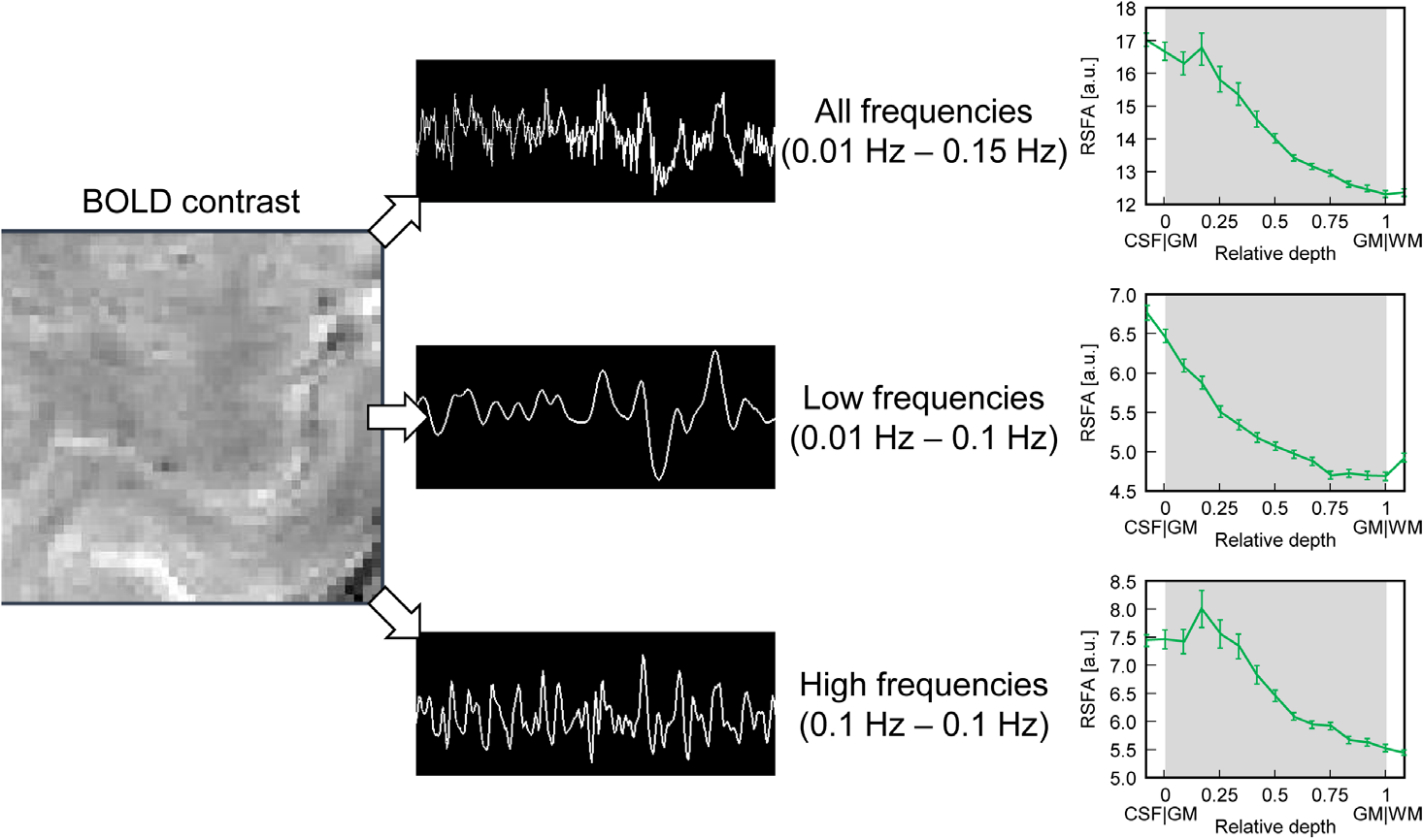
Schematic illustrating the computation of resting‐state fluctuation amplitudes in one representative participant. A high‐pass cutoff of 0.01 Hz is first applied to all resting‐state blood oxygenation level‐dependent (BOLD) signal timeseries. The timeseries is additionally bandpass filtered into a low‐frequency band (0.01–0.1 Hz) and a high‐frequency band (0.1–0.15 Hz). Resting‐state fluctuation amplitude (RSFA) values are then computed as temporal *SDs* of the corresponding timeseries for the entire frequency band (a), the low‐frequency band (b), and the high‐frequency band (c)

Following Kannurpatti, Rypma, and Biswal (2012), the resting‐state fluctuation amplitudes of the unfiltered timeseries (RSFA_full_) as well as of their low‐frequency (RSFA_low_) and high‐frequency (RSFA_high_) portions were taken to be the corresponding temporal standard deviation (tSD_rs_). The low‐frequency and high‐frequency timeseries included frequencies in the bands between 0.01 and 0.1 Hz and between 0.1 and 0.15 Hz, respectively. The unfiltered timeseries included frequencies in the entire range between 0.01 and 0.15 Hz as defined by scan duration and TR. All RSFA values were calculated for each lamina (Figure 2). Similarly, the amplitude of hypercapnia‐induced signal changes was taken to be the temporal standard deviation of the corresponding timeseries (tSD_hc_) (Kannurpatti & Biswal, 2008).

The calculation of the calibration factor, *M*, followed the approach presented in previous work (Guidi et al., 2016). In brief, the Davis model (Davis et al., 1998; Hoge et al., 1999) was modified to express BOLD signal changes in terms of CBV and CMR_**O2**_ and to account for the CBF term using Grubb's relationship (Grubb, Raichle, Eichling, & Ter‐Pogossian, 1974). The original Davis model can be written as

(1) $$\Delta S=M∙\left[1-f^{\alpha_{t}-\beta }∙r^{\beta }\right].$$

Δ*S* = (*S* − *S*_0_)/*S*_0_ is the relative BOLD signal change from the baseline level (indicated by an index “0”); *f* = CBF/CBF_0_ and *r* = CMR_O2_/CMR_**O**2, 0_ are the normalized cerebral blood flow and oxidative metabolic rate, respectively; and *α*_*t*_ = 0.38 is the Grubb exponent relating the total blood volume (CBV_*t*_) to CBF. *β* is a magnetic field‐specific constant describing the coupling between the reversible transverse relaxation rate, $R_{2}^{\prime }$, induced by inhomogeneous external fields and [dHb]. In general, it depends on the vessel size but may be approximated as *β* ≈ 1 at 7 T, where intravascular signal contributions become negligible at typical TE values (Bright, Croal, Blockley, & Bulte, 2019; Kida, Kennan, Rothman, Behar, & Hyder, 2000; Martindale, Kennerley, Johnston, Zheng, & Mayhew, 2008). Consequently, the change in $R_{2}^{\prime }$ is approximately linear in [dHb]. *M* represents the maximum possible BOLD signal change and depends on TE and the baseline levels of venous blood volume (CBV_*v*_) and [dHb] in addition to further parameters (e.g., vessel size, brain region, magnetic field strength), which may be lumped together into a proportionality constant *κ*:

(2) $$M=\kappa ∙\mathrm{TE}∙{\mathrm{CBV}}_{v,0}∙{\left[\mathrm{dHb}\right]}_{0}^{\beta }.$$

In the modified Davis model, the dependency on *f* in Equation (1) is replaced by a dependency on *v*_*t*_ = CBV_*t*_/CBV_*t*, 0_, yielding (Guidi et al., 2016):

(3) $$\Delta S=M\left[1-v_{t}^{\left(\alpha_{v}-\beta \right)/\alpha_{t}}∙r^{\beta }\right].$$

Here, *α*_*v*_ = 0.2 is a modified Grubb exponent relating CBV_*v*_ to CBF (Chen & Pike, 2009). For mild hypercapnia assumed to be isometabolic, *r* ≈ 1, and the evoked BOLD signal change can be expressed as:

(4) $$\Delta S_{\mathrm{hc}}=M\left[1-v_{t}^{\left(\alpha_{v}-\beta \right)/\alpha_{t}}\right].$$

**Figure 3:**
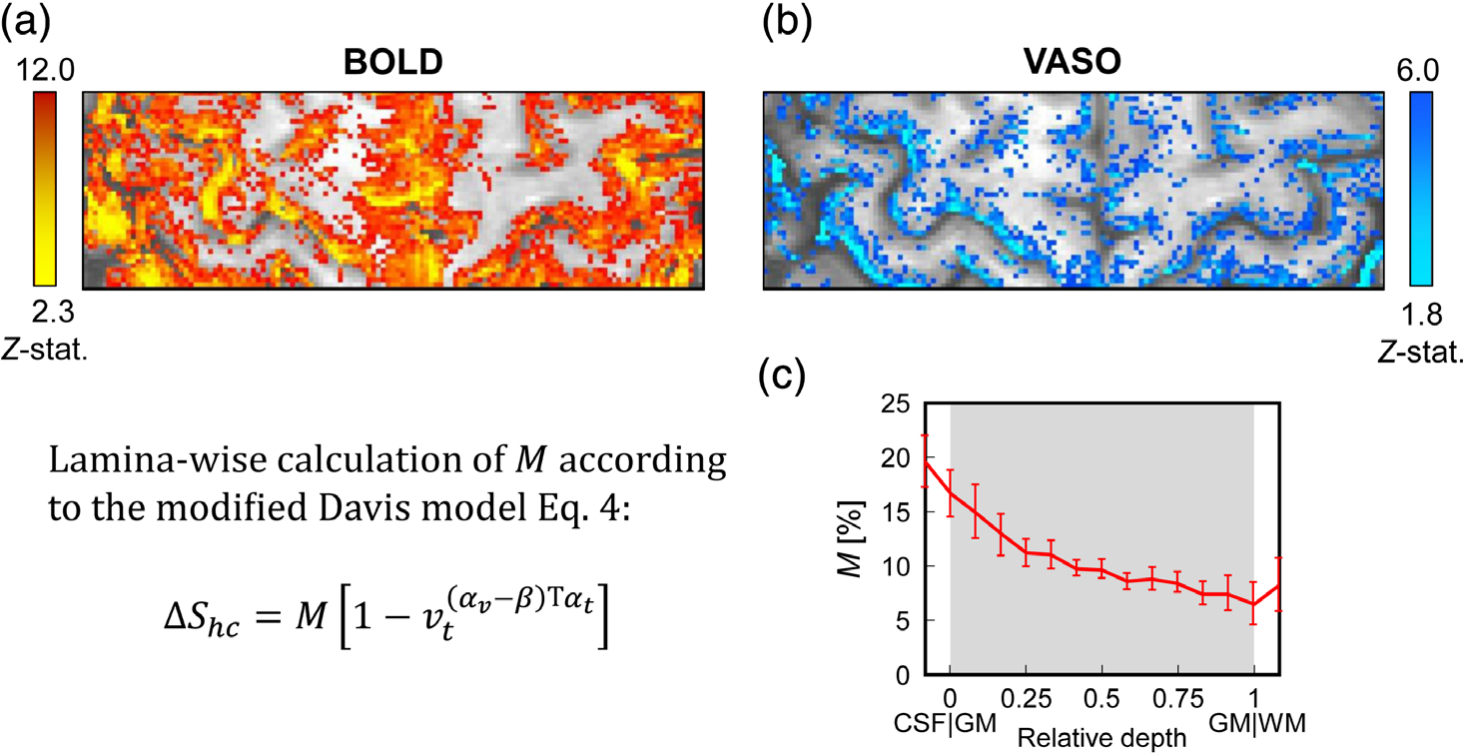
*Z*‐stat maps of hypercapnia‐induced blood oxygenation level‐dependent (BOLD) (a) and vascular space occupancy (VASO) (b) signal changes and calculation of the calibration parameter *M* (c). Error bars refer to the *SE* of the mean. An region of interest (ROI) encompassing the hand‐knob area of the left hemisphere with significant BOLD (*Z* > 2.3; *p* < .01) and VASO signal changes (*Z* > 1.8; *p* < .05) was chosen for the calculation of *M* using Equation (4)

If laminar BOLD and CBV changes are recorded during a hypercapnic challenge, *M* is easily obtained with Equation (4) for each lamina (Figure 3).

**Figure 4:**
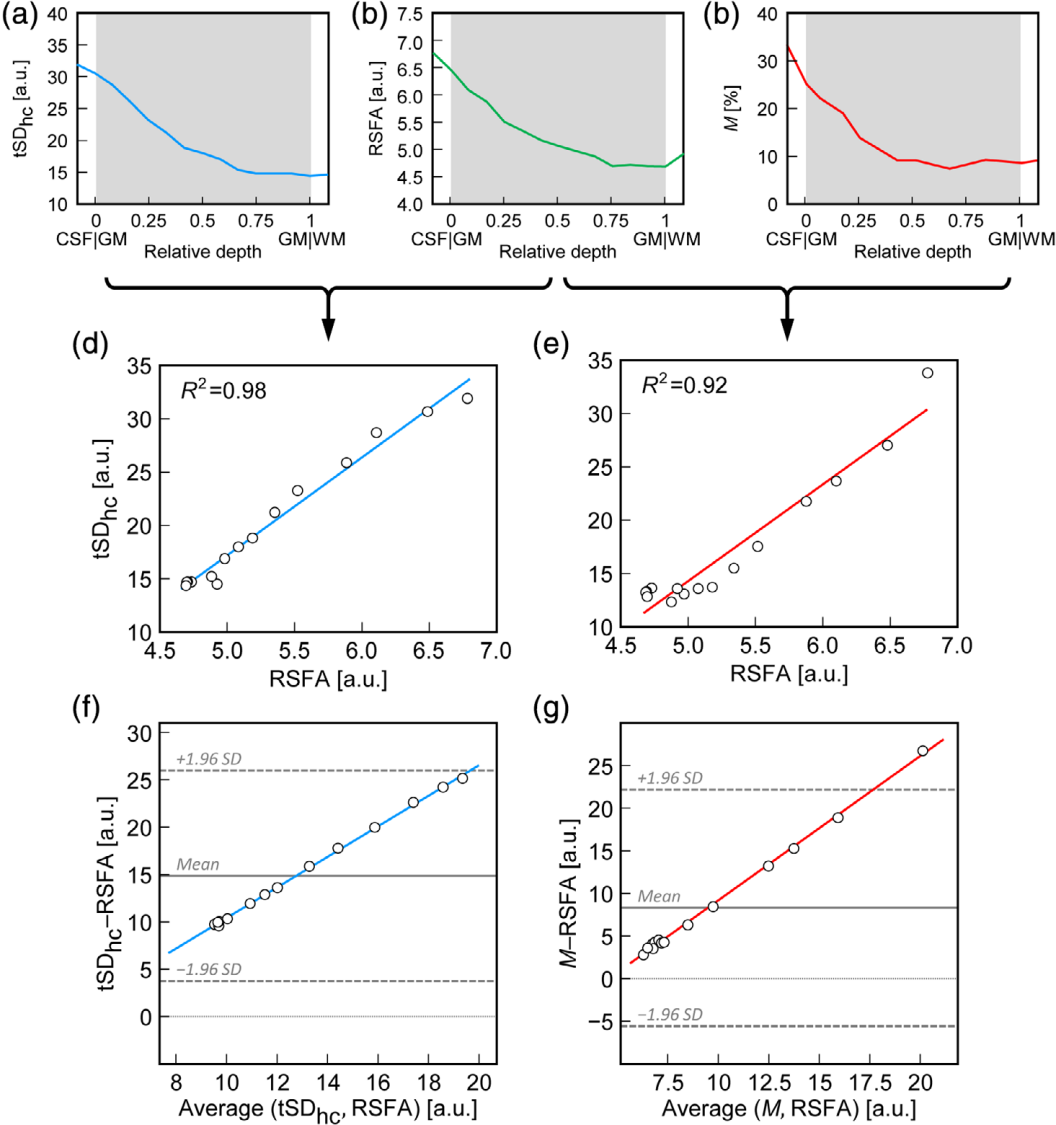
Example of linear regression construction for one representative participant. The laminar values of tSD_hc_ **(**a) and *M* (c) are plotted against laminar RSFA_full_ (b). The linear regression results for tSD_hc_ versus RSFA_full_ (d) and *M* versus RSFA_full_ (e) are also shown. Each point corresponds to a single lamina. Corresponding Bland–Altman diagrams of the same comparisons (f, g) indicate proportional constant (i.e., linear) behavior. Horizontal solid gray lines indicate mean differences (in the observed range), and horizontal dashed gray lines indicate a range of ±1.96 *SDs*

The depth‐dependent values of *M* and tSD_hc_ from individual participants were correlated separately with each of the RSFA profiles. Scatter plots of the obtained correlations were then constructed where each point represents one lamina, and the squared Pearson correlation coefficient (*R*^2^) for the linear regression was computed (Figure 4).

### 2.2 Comparison of RSFA‐normalized BOLD and CBV laminar profiles

Additional data were available from previous experiments at the National Institutes of Health (NIH) investigating the cortical input and output in human M1 (see https://layerfmri.page.link/RSFA_data). Full details of these acquisitions and image‐processing procedures have been published by Huber et al. (2017). The data had been acquired employing similar methods as in part 1 of this work under an NIH Combined Neuroscience Institutional Review Board‐approved protocol (93‐M‐0170, ClinicalTrials.gov identifier: NCT00001360) in accordance with the Belmont Report and US Federal Regulations that protect human subjects. In particular, SS‐SI‐VASO timeseries had been obtained in 11 healthy, right‐handed subjects (6 males; age 23–43 years) with TR = 1.5 s and a nominal voxel size of 0.71 × 0.71 × 1.5 mm^3^. Analysis of the performance of RSFA‐based normalization included a 24‐min block of left‐hand finger tapping without touch and a 24‐min resting‐state block. Measures of low frequency fluctuations were extracted from (a) the resting‐state data and (b) from the residuals of the tapping data, and RSFA was estimated in FMRIB Software Library (FSL) (Jenkinson, Beckmann, Behrens, Woolrich, & Smith, 2012) and AFNI (Cox, 1996). Subsequently, 21 laminae were estimated with LAYNII (https://github.com/layerfMRI/LAYNII) in M1.

## 3 RESULTS

### 3.1 Comparison of RSFA‐based normalization and gas calibration

All participants tolerated the breathing manipulation well. One participant was excluded from the final analysis due to excessive motion (amplitude exceeding 1 mm) during the gas manipulation session. Accommodation of up to 15 equivolume laminae within the cortical ribbon of the hand‐knob area was achieved in all participants. For the hypercapnia challenge, the tSNR measured in the ROI was between 25 and 35 for the BOLD and between 15 and 25 for the VASO timeseries.

All RSFA and tSD_hc_ profiles showed larger values at the cortical surface and gradually decreased towards the WM. The mean depth‐dependent profile of *M* showed a similar decreasing pattern (Figure 4), however, with increased variation between participants.

All linear regression analyses yielded positive correlations. The correlations of RSFA and tSD_hc_ were significant (*p* < .01) in all participants, with *R*^2^ = 0.92 ± 0.06 (mean ± 1 *SD* across participants) for RSFA_full_, 0.94 ± 0.06 for RSFA_low_, and 0.92 ± 0.08 for RSFA_high_ (Table 1). For the correlations of RSFA and *M*, *R*^2^ was 0.62 ± 0.19 (*p* < .05 in 8 out of 9 participants) for RSFA_full_, 0.60 ± 0.26 (*p* < .05 in 7 out of 9 participants) for RSFA_low_, and 0.55 ± 0.26 (*p* < .05 in 7 out of 9 participants) for RSFA_high_ (Table 2). Of note, applying RETROICOR correction did not substantially affect these correlations. This is consistent with previous research demonstrating that corrections methods attempting to minimize contributions of physiological noise become less relevant at high (e.g., submillimeter) resolution, where noise is increasingly dominated by thermal fluctuations (Hall et al., 2017; Murphy, Bodurka, & Bandettini, 2007).

**Table 1:** Individual correlation coefficients for linear regressions of resting‐state fluctuation amplitude (RSFA) and tSD_hc_

Participant	*R*^2^		
	tSD_hc_ versus RSFA_full_	tSD_hc_ versus RSFA_low_	tSD_hc_ versus RSFA_high_
1	0.79	0.81	0.86
2	0.87	0.95	0.98
3	0.90	0.89	0.83
4	0.89	0.92	0.89
5	0.92	0.98	0.78
6	0.98	0.99	0.98
7	0.96	0.96	0.98
8	0.99	0.99	0.99
9	0.96	0.95	0.98
Group	0.92 ± 0.06	0.94 ± 0.06	0.92 ± 0.08

*Note*: Frequency bands were 0.01–0.15, 0.01–0.1, and 0.1–0.15 Hz for RSFA_full_, RSFA_low_, and RSFA_high_, respectively. Group mean values and *SDs* across participants are also included.

**Table 2:** Individual correlation coefficients for linear regressions of resting‐state fluctuation amplitude (RSFA) and *M*

Participant	*R*^2^		
	*M* versus RSFA_full_	*M* versus RSFA_low_	*M* versus RSFA_high_
1	0.48	0.57	0.62
2	0.61	0.28	0.23
3	0.82	0.80	0.75
4	0.38	0.19	0.14
5	0.70	0.93	0.50
6	0.46	0.50	0.51
7	0.74	0.74	0.84
8	0.92	0.90	0.92
9	0.44	0.46	0.46
Group	0.62 ± 0.19	0.60 ± 0.26	0.55 ± 0.26

*Note*: Frequency bands were 0.01–0.15, 0.01–0.1, and 0.1–0.15 Hz for RSFA_full_, RSFA_low_, and RSFA_high_, respectively. Group mean values and *SDs* across participants are also included.

Additional Bland–Altman analyses of the correlations of tSD_hc_ and *M* with RSFA (Figure 4c) indicate proportional constant bias (linear slope) for both correlations with very subtle constant variability. The linear behavior is expected if all measures depend on a common physiological mechanism (i.e., the local intrinsic vascular sensitivity), which is, however, assessed under different experimental conditions yielding numerically different scaling variables. This is in line with results from Kannurpatti and Biswal (2008) demonstrating different extents of scaling but similar reductions in the variance of the BOLD response upon hemodynamic scaling based on RSFA, a breath‐holding task, or a CO_2_ challenge.

### 3.2 Comparison of RSFA‐normalized BOLD and CBV laminar profiles

**Figure 5:**
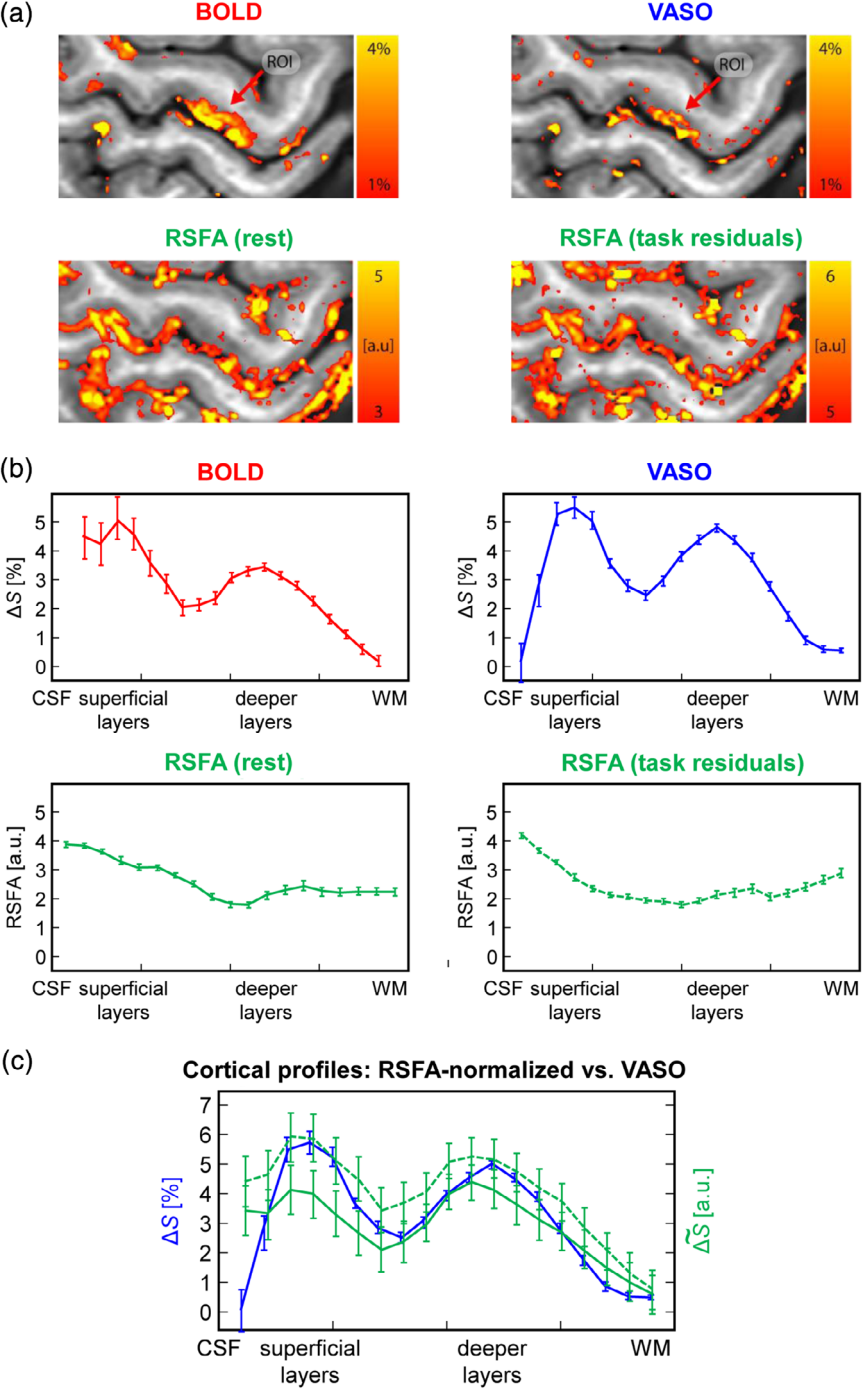
Comparison of resting‐state fluctuation amplitude (RSFA)‐normalization of blood oxygenation level‐dependent (BOLD) signal changes on a laminar level to results from vascular space occupancy (VASO)‐based cerebral blood volume (CBV) measurements. (a) Color‐coded maps of BOLD (top left) and VASO (top right) percent signal changes and maps of RSFA extracted from resting‐state time series (bottom left) and from the residuals of task‐based time series (bottom right). The gray‐scale background corresponds to the inherently *T*_1_‐weighted signal of the functional time series yielding excellent contrast between gray matter (GM) and white matter (WM). Note that comparable results are obtained with both RSFA maps. (b) Corresponding laminar profiles through M1 in the region of interest (ROI) indicated in (a). (c) Laminar BOLD profiles after RSFA‐based normalization (shown in green color) in comparison to the VASO profile (shown in blue color). Solid and dashed green lines indicate normalizations with RSFA from the resting‐state time series and from the residuals of the task‐based time series, respectively. Similar results are obtained with both RSFA‐profiles

**Figure 6:**
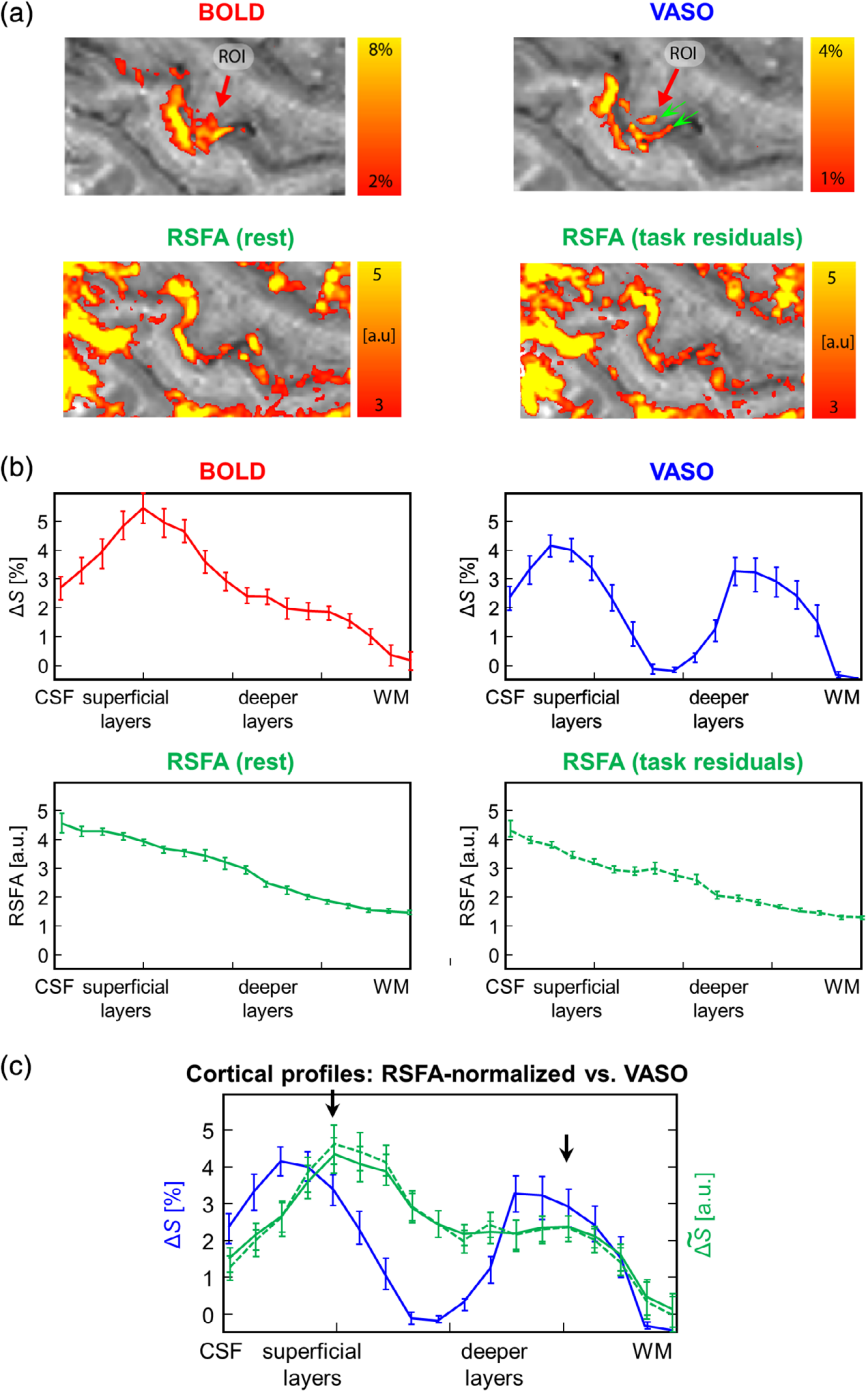
The same type of comparison as in Figure 5 in a participant without a clear double‐peak pattern in the laminar blood oxygenation level‐dependent (BOLD) response. (a) Color‐coded maps of BOLD (top left) and vascular space occupancy (VASO) (top right) percent signal changes and maps of resting‐state fluctuation amplitude (RSFA) extracted from resting‐state time series (bottom left) and from the residuals of task‐based time series (bottom right). (b) Corresponding laminar profiles through M1 in the region of interest (ROI) indicated in (a). (c) Laminar BOLD profiles (green lines) after RSFA‐based normalization in comparison to the VASO profile (blue solid line). Solid and dashed green lines indicate normalizations with RSFA from the resting‐state time series and from the residuals of the task‐based time series, respectively. Similar results are obtained with both RSFA‐profiles

Selected maps of tapping‐induced percent BOLD and VASO signal changes and of the RSFA extracted from the resting‐state and from the residuals of the tapping data as well as corresponding laminar profiles in M1 are shown in Figures 5 and 6. The laminar CBV profile in Figure 5b shows a clear indication of a double‐peak pattern, consistent with previous reports (Huber et al., 2015; Huber et al., 2017). The corresponding laminar BOLD profile demonstrates signal amplification in superficial laminae due to venous drainage (Turner, 2002). A pronounced amplitude increase towards the pial surface is also evident in both RSFA profiles. Figure 5c shows RSFA‐normalized laminar BOLD profiles in direct comparison to the CBV profile from Figure 5b indicating a slightly improved depiction of the peak in upper layers. We note, however, that laminar fMRI responses can be quite variable across participants. As such, while the double peak was robustly visible in previous VASO data, only one third of the participants showed the same feature in the BOLD response (Huber et al., 2017). To further exemplify the working principle of the RSFA method, we illustrate a corresponding case without a double‐peak BOLD pattern in Figure 6. In this participant, VASO data show separable activity in superficial and deeper layers of M1 (green arrows), whereas the BOLD response yields one condensed “blob” (Figure 6a). As in Figure 5a, the RSFA maps show strongest values in superficial layers and in the pial vasculature (Figure 6b), and RSFA normalization reduces the slope of the BOLD profiles. As a result, subtle slope variations across the cortical depth in the original BOLD profile are enhanced indicating separate peaks or shoulders (black arrows in Figure 6c).

## 4 DISCUSSION

Nearly perfect correlations of RSFA and tSD_hc_ were found for all examined frequency bands. This confirms previous results obtained on a voxel‐wise level at standard fMRI resolution (Kannurpatti & Biswal, 2008). Our correlation strengths were somewhat higher than those reported by Kannurpatti et al. (2012). This may be attributed to the fact that linear regression was performed on a laminar basis in our work, that is, on more local level and with substantial blurring across laminae due to the relatively high degree of interpolation in this direction. There were no significant differences in the correlation strengths when comparing the high‐ and low‐frequency bands, indicating similar scaling along the cortical depth. This assumption is further corroborated by the good agreement of laminar RSFA‐normalized BOLD profiles and VASO profiles (Figure 5c).

Significant positive correlations were also found between RSFA and *M*. As for the correlations with tSD_hc_, similar correlation strengths were obtained for the different frequency bands in the majority of the participants. *M* can be considered in our work to be only modulated by subject‐specific parameters, since sequence‐specific parameters were identical in all acquisitions (see Equation (2)). This is not the case for RSFA, which does not entirely depend on the baseline levels but also on dynamic changes of CBF and CBV. Thus, we cannot exclude that lower correlations (relative to the above results for tSD_hc_) might be partially due to the different physiological modulators of RSFA with respect to *M*. However, reduced correlations are also expected due to the variability of the *M* parameter, which is computed by a division of relatively subtle BOLD and CBV changes and is, hence, inherently noisier than a pure contrast.

The low‐ and high‐frequency bands as defined here showed similar scaling as a function of depth. The low‐frequency band (0.01–0.1 Hz) is usually considered to be the one reflecting the relevant neuronal activity (Biswal, Yetkin, Haughton, & Hyde, 1995; Margulies et al., 2010). However, it is obvious from our laminar profiles (Figure 2) that even this band is dominated by contributions from the superficial draining vasculature, which does not correspond to the precise location of neuronal activity (Turner, 2002). While this does not rule out the possibility that the neuronal component is present in this band, it is likely to add some confounds to it (Markuerkiaga et al., 2016). The high frequency band (0.1–0.15 Hz) selected in our analysis is narrower than the traditional high‐frequency band (0.1–0.25 Hz), whose definition is based on typical fMRI studies with TR ≈ 2 s. Similar to the results observed for the low‐frequency band, the high‐frequency band is also dominated by the pial surface, therefore, yielding almost identical correlations. Given the spatial nature of pial vein contaminations, it is reasonable to expect that the correlation would be disrupted as soon as its relative contribution becomes negligible, and the shape of the depth‐dependent RSFA starts flattening out. This effect was visible at frequencies above 0.15 Hz (Guidi et al., 2017) and is also consistent with the profiles shown in Figures 5b and 6b.

Based on our results, RSFA seems to provide a good substitute for the amplitude of hypercapnia‐induced signal changes to scale the BOLD response. Regarding the assumption of an isometabolic hypercapnia challenge, RSFA and tSD_hc_ are likely to be similarly driven by reactivity features of the underlying vasculature (both following a relation similar to Equation (1)) and, therefore, look like scaled versions of each other.

Despite the good agreement of RSFA and *M*, a similar statement cannot be made in this case. This is due to their different physiological origins, with *M* depending solely on baseline parameters as long as it is accurately measured (Griffeth & Buxton, 2011). In fact, a strict correlation of RSFA and *M* might be not as straightforward as it seems (Lu, Hutchison, Xu, & Rypma, 2011). The calculation of *M* relies on several assumptions, whose validity is called into question, especially at high resolution. Such potential pitfalls include (among others) the validity of a Grubb‐like relation between CBF and CBV with a suitable coupling exponent *α*, the assumption of an isometabolic hypercapnia challenge, or the validity of Fick's principle on a laminar basis. More extended discussions of these issues have been published recently (Guidi et al., 2016; Hua et al., 2019; Huber et al., 2019). For example, simulations assuming different values for α and β indicate that these variations primarily produce a scaling effect, whereas the laminar profiles were largely preserved (see Supplementary Information in [Guidi et al., 2016]). This seems to indicate that these factors should not critically affect our main conclusions.

The application of fMRI signal normalization along the cortical depth may further be associated with limited interpretability in a more general way:

- We have shown in earlier work (Huber et al., 2015) that—in the process of normalization using depth‐dependent physiological parameters—activity in some cortical layers may be undesirably underestimated. In particular, higher values of, for example, cerebrovascular reactivity (CVR), CBV_0_, or CBV_*v*,0_ in upper cortical layers may lead to artificially reduced normalized fMRI signal changes in these layers, despite the fact that the microvascular‐related response has a similar magnitude (see also figure 8 from Huber et al. (2015)).
- When the fMRI signal is normalized, the resulting activity measure depends on a number of estimated parameters as opposed to non‐normalized activity measures. Due to nonlinear error propagation of multiple parameters during the normalization, this might result in a noisier normalized activity measure.
- In the current work, we investigated the possibility of fMRI signal normalization by means of dividing fMRI responses by various measures of signal variability. Such divisions are nonlinear operations and may reshape the cortical profiles, potentially generating unfamiliar profiles, which may be harder to interpret. As such, in voxels where the CVR is very low and, hence, noisy (e.g., for large partial voluming with WM), the denominator can become very small, which may lead to an unphysiologically large normalized fMRI signal.

Scaling approaches, such as measures of the CVR Liu et al., 2013; Liu et al., 2017, which rely on normalization with BOLD‐based perfusion‐sensitive scans but without additional CBF or CBV recordings cannot deliver estimates of CMR_O2_ changes. This also applies to normalization with RSFA as investigated here. For certain applications, however, including studies at submillimeter resolution or high field (i.e., 7 T and beyond), the traditional way for extracting information about CMR_O2_ with a separate measurement of CBF changes might be difficult to achieve with a sufficient tSNR. Such applications may not be compromised by a missing possibility to quantify CMR_O2_ but would still benefit from calibrations employing RSFA maps.

To further evaluate the quality of laminar profiles obtained after RFSA‐based scaling, we used results obtained with VASO (i.e., a measure of the CBV response) as a reference of high spatial specificity. In general, the RFSA‐normalized laminar BOLD profiles agreed well with the VASO results achieving a subtle improvement in the depiction of the expected double‐peak contour (Figures 5c and 6c). This suggests that the normalization accounts for venous bias inherent to the BOLD response with respect to signal amplification. It is to note that quite similar results were obtained with both RSFA estimates (i.e., from the task‐based time series and from an additional resting‐state time series) indicating that sufficient information can be extracted directly from the task‐based fMRI time series. However, some deviation remained, which was most pronounced at the pial surface pointing to the fact that voxel‐wise RSFA‐normalization does not account for local signal leakage.

Recent modeling results indicate that the baseline CBV variation due to the arrangement of ascending veins rather than venous draining alone contributes to the amplitude increase of the laminar BOLD response (Havlicek & Uludağ, 2020; Markuerkiaga et al., 2016). The RSFA normalization as described here is a *linear* scaling approach that may be considered to correct—at first order—for local signal amplification in layers with higher CVR, However, such scaling does not account for signal leakage due to venous drainage from the location of distant layers. In particular, the laminar point‐spread function of the BOLD response is not entirely defined by the local baseline CBV but depends *nonlinearly* on multiple physiological factors and on the level of activity (Havlicek & Uludağ, 2020; Huber et al., 2014a). Future development will be necessary to incorporate higher‐order normalization of signal leakage. As such, an integration of RSFA factors as proposed here into laminar‐deconvolution models (Havlicek & Uludağ, 2020; Heinzle, Koopmans, den Ouden, Raman, & Stephan, 2016; Markuerkiaga et al., 2016; Merola & Weiskopf, 2018) might be an interesting topic of future research. Such models currently rely on assumptions on cortical depth‐dependent fMRI reactivity (e.g., from ex‐vivo data), which are not easily generalizable across brain areas beyond the primary visual cortex (V1) (Marquardt, Schneider, Gulban, Ivanov, & Uludağ, 2018). In this context, the RSFA method might provide a data‐driven means for venous bias correction in laminar fMRI across brain areas and participants.

Other researchers tried to account for venous bias through linear postprocessing strategies, for example, by regressing out a linear slope of the laminar GRE‐BOLD profile as a zeroth‐order correction (Fracasso, Petridou, & Dumoulin, 2014) or by a decomposition of the profile into a constant and a linear term (Gau, Bazin, Trampel, Turner, & Noppeney, 2020). Similar to the RSFA method, these concepts are based on some form of linear scaling. Unlike the RSFA method, however, they are limited by the fact that the correction factors are not determined form a temporally orthogonal fMRI signal, which might introduce some level of circularity into the analysis. Moreover, these methods cannot account for scaling effects that have nonconstant slopes. We may, therefore, speculate that the RSFA approach could be useful for accounting for additional sources of venous scaling compared to linear slope methods.

As another strategy, Muckli et al. (2015) proposed to account for venous scaling bias by means of constraining the fMRI activity interpretation to statistical measures of “classification accuracy.” Unlike the BOLD signal magnitude, this measure is inversely proportional to the signal variance, which in turn is dependent on CVR. Thus, the measure of ‘classification accuracy’ should be inherently weighted by a CBV‐dependent scale factor, which is comparable to RSFA‐based normalization. Lawrence et al. (2018) used a similar statistical scaling by means of layer‐dependent *t*‐scoring. It is argued that this normalizes the BOLD signal change by the vein‐dependent signal variance (similar to assumptions underlying RSFA normalization). However, these methods are not exclusively specific to CVR‐related signal variance but might be also affected by high‐frequency signal fluctuations, including variance from thermal noise and CSF motion. In this sense, the RSFA approach might be a more accurate measure of vasculature‐driven signal variance.

## 5 CONCLUSIONS

We have shown that the laminar amplitude of spontaneous brain fluctuations resembles almost perfectly the scaling of the laminar amplitude of hypercapnia‐induced signal changes, which are frequently used as a biomarker of CVR. This result points to the fact that RSFA measures can be used to replace tSD_hc_ as a scaling factor for normalizing the BOLD response. Despite its different physiological origin, the calibration parameter *M* also showed remarkable similarities with the RSFA profile. The shape of laminar BOLD signal changes reflects spatial variations in baseline CBV_*v*_, which explains the similarities observed for the laminar profiles of *M*, tSD_hc_, and RSFA, and the consequently strong correlations.

## CONFLICT OF INTEREST

The authors declare no conflict of interest.

## Data Availability

The data that support the findings of this study are available from the corresponding author upon reasonable request.
